# Nicotinamide Riboside-Driven Modulation of SIRT3/mtROS/JNK Signaling Pathways Alleviates Myocardial Ischemia-Reperfusion Injury

**DOI:** 10.7150/ijms.97530

**Published:** 2024-08-12

**Authors:** Lingqing Wang, Changgong Chen, Hao Zhou, Luyuan Tao, Enguo Xu

**Affiliations:** 1 Department of Cardiovascular Internal Medicine, Taizhou First People's Hospital, Wenzhou Medical University, Zhejiang, China.; 2 School of Medicine, Southern University of Science and Technology, Shenzhen, Guangdong, China.

**Keywords:** Nicotinamide Riboside, SIRT3, mtROS, JNK, cardiac ischemia reperfusion injury.

## Abstract

Myocardial ischemia-reperfusion (I/R) injury exacerbates cellular damage upon restoring blood flow to ischemic cardiac tissue, causing oxidative stress, inflammation, and apoptosis. This study investigates Nicotinamide Riboside (NR), a precursor of nicotinamide adenine dinucleotide (NAD^+^), for its cardioprotective effects. Administering NR to mice before I/R injury and evaluating heart function via echocardiography showed that NR significantly improved heart function, increased left ventricular ejection fraction (LVEF) and fractional shortening (FS), and reduced left ventricular end-diastolic (LVDd) and end-systolic diameters (LVSd). NR also restored E/A and E/e' ratios. It reduced cardiomyocyte apoptosis both *in vivo* and *in vitro*, inhibiting elevated caspase-3 activity and returning Bax protein levels to normal. *In vitro*, NR reduced the apoptotic rate in hydrogen peroxide (H2O2)-treated HL-1 cells from 30% to 10%. Mechanistically, NR modulated the SIRT3/mtROS/JNK pathway, reversing H2O2-induced SIRT3 downregulation, reducing mitochondrial reactive oxygen species (mtROS), and inhibiting JNK activation. Using SIRT3-knockout (SIRT3-KO) mice, we confirmed that NR's cardioprotective effects depend on SIRT3. Echocardiography showed that NR's benefits were abrogated in SIRT3-KO mice. In conclusion, NR provides significant cardioprotection against myocardial I/R injury by enhancing NAD+ levels and modulating the SIRT3/mtROS/JNK pathway, suggesting its potential as a novel therapeutic agent for ischemic heart diseases, meriting further clinical research.

## Introduction

Myocardial ischemia-reperfusion (I/R) injury remains a significant clinical challenge in the treatment of ischemic heart diseases 1. The paradoxical exacerbation of cellular damage upon the restoration of blood flow to ischemic cardiac tissue leads to oxidative stress, inflammation, and apoptotic cell death, thereby limiting the benefits of reperfusion therapies 2, 3. Despite advancements in therapeutic interventions, the incidence of heart failure and adverse cardiac events post-reperfusion remains high 4-7. Therefore, understanding the molecular mechanisms underlying I/R injury and developing novel therapeutic strategies to mitigate this damage are of paramount importance for improving patient outcomes.

Nicotinamide Riboside (NR) is a naturally occurring form of vitamin B3 and a precursor to nicotinamide adenine dinucleotide (NAD^+^), a crucial coenzyme in cellular metabolism 8. NR has garnered attention for its potential to enhance NAD^+^ levels, which decline with age and stress, thereby influencing various metabolic and repair processes 9. Recent studies have demonstrated the cardioprotective effects of NR, particularly in the context of myocardial I/R injury 10. By boosting NAD^+^ levels, NR activates sirtuins, especially SIRT3, which plays a key role in maintaining mitochondrial function and reducing oxidative stress 11-13. The ability of NR to modulate mitochondrial reactive oxygen species (mtROS) production and improve mitochondrial bioenergetics positions it as a promising therapeutic candidate for cardiovascular diseases 14, 15.

SIRT3, a mitochondrial deacetylase, is crucial for maintaining mitochondrial integrity and function 16, 17. It enhances the activity of antioxidant enzymes, thereby reducing mtROS production, a major contributor to I/R-induced oxidative damage 9, 18, 19. The JNK (c-Jun N-terminal kinase) signaling pathway is activated in response to stress, including oxidative stress during I/R injury, leading to inflammation and apoptosis 20, 21. Emerging evidence suggests that SIRT3 can negatively regulate the JNK pathway, thus attenuating its deleterious effects 22-24. The interplay between SIRT3, mtROS, and JNK signaling forms a complex regulatory network that is pivotal in the pathogenesis of myocardial I/R injury. Understanding these interactions offers potential therapeutic targets for mitigating I/R-induced cardiac damage.

In summary, myocardial I/R injury poses a significant clinical challenge, necessitating the exploration of novel therapeutic approaches. Nicotinamide Riboside, by enhancing NAD^+^ levels, offers a promising strategy to activate protective pathways involving SIRT3, mtROS, and JNK signaling. This article aims to investigate the modulatory effects of NR on these pathways and its potential in alleviating myocardial I/R injury. By elucidating the underlying mechanisms, this study seeks to contribute to the development of effective treatments for ischemic heart diseases.

## Methods

### Ethical statement

This study adhered to the principles of the Declaration of Helsinki and was conducted in compliance with the ethical guidelines of Wenzhou Medical University. The research protocol received ethical approval from the university's Ethics Committee, with the approval number WZMU-2023-03W.

### Myocardial Ischemia and Reperfusion

To establish the myocardial ischemia-reperfusion (I/R) injury model, we used Wild-Type (WT) C57BL/6 mice and SIRT3 knockout (SIRT3-KO, Strain #:027975, Jackson Laboratory) mice, all aged 10 weeks. The mice were randomized and anesthetized using isoflurane inhalation. They were then endotracheally intubated and placed on a rodent ventilator. The left anterior descending (LAD) coronary artery was exposed and occluded with a prolene suture for 45 minutes following the removal of the pericardium. Successful occlusion was confirmed by the whitening of the left ventricle region. The suture was then released to allow reperfusion. Mice with a left ventricular ejection fraction (LVEF) between 55-60% one day post-occlusion were considered to have a successful I/R model. Prior to I/R injury, mice were administered Nicotinamide Riboside (NR, NIAGEN® ChromaDex) at doses ranging from 200-800 mg/kg/day diluted in drinking water for seven days 25.

### Echocardiography

Cardiac function and dimensions were assessed *in vivo* using a high-resolution 2D echocardiography system (Esaote MyLab Twice, Italy). Light anesthesia was maintained using isoflurane (#100150, Shanghai Yuyan Instruments Co. Ltd) to minimize heart rate reduction. The interventricular septum (IVS), left ventricular posterior wall thickness (LVPW), and left ventricular internal dimension (LVID) were measured at both diastole and systole from M-mode images. End-diastole was defined as the maximal LV diastolic dimension, while end-systole was identified as the most anterior systolic excursion of the LV posterior wall. LVEF was calculated as (EDV - ESV) / EDV × 100%, where EDV is the end-diastolic volume and ESV is the end-systolic volume. Left ventricular fractional shortening (LVFS) was computed as (LVIDd - LVIDs) / LVIDd × 100%.

### Caspase 3 activity measurement

Caspase 3 activity in cells and tissues was measured using the Caspase 3 Colorimetric Assay Kit (Biovision) according to the manufacturer's protocol. The activity was normalized to total protein levels to ensure accurate quantification 26.

### MitoTracker Immunofluorescence Staining

HL-1 cells were seeded onto 8-well chamber slides and incubated with 50nM MitoTracker Red CMXRos (Cell Signaling #9082) for 45 minutes at 37°C. Post-incubation, the cells were washed with 1X PBS and fixed in ice-cold methanol at -20°C for 15 minutes. The cells were then washed three times with PBS and blocked with 10% donkey serum for 1 hour at room temperature. Cells were incubated with cTnT (R&D System #MAB1874, 1:200) overnight at 4°C, followed by incubation with Alexa Fluor 488 secondary antibody for 1 hour at room temperature. Nuclear staining was performed using DAPI. Images were captured using an Olympus IX83 confocal microscope. Mitotracker signal intensity was measured using ImageJ software, and differences between groups were analyzed statistically 27.

### Quantitative Real-Time PCR

Total RNA was extracted from frozen heart tissue using the RNeasy Mini kit (Qiagen), and cDNA synthesis was performed using reverse transcriptase. Quantitative PCR was conducted using SYBR Green Master Mix (Roche) under a 40-cycle thermocycling protocol. The ΔΔCT method was used to represent relative mRNA levels normalized to housekeeping genes 28.

### Western blot analyses

Whole cell lysates were prepared from HL-1 cells using T-PER buffer (Fisher Scientific) supplemented with Halt™ protease and phosphatase inhibitors (Thermo Fisher). Lysates were clarified by centrifugation at 10,000g for 20 minutes at 4°C, and loading buffer was added to the supernatant before denaturation by boiling for 5 minutes. Denatured lysates were sonicated prior to loading onto SDS-PAGE gels. Equal amounts of protein (20 µg for cells, 50 µg for tissues) were separated by 10% SDS-PAGE and transferred to PVDF membranes (Millipore). Membranes were probed with HRP-conjugated secondary antibodies at a 1:1000 dilution and visualized using ECL reagent (Pierce). Signal intensity of both phosphorylated and total proteins was quantified using ImageJ software 29.

### ELISA

To assess JNK activity, cultured cells were homogenized in lysis buffer containing protease and phosphatase inhibitors. Protein concentrations were determined using the Bradford assay. An ELISA kit specific for JNK activity was used according to the manufacturer's instructions. Equal protein amounts were added to ELISA plate wells pre-coated with JNK antibodies and incubated 30. After washing, a detection antibody specific for phosphorylated JNK was added, followed by another incubation and wash. Substrate solution was then added, and color change was measured using a microplate reader. Absorbance values were compared to a standard curve to quantify JNK activity. The same procedure was followed for measuring Bax activity 31.

### TUNEL Analysis

Cell death in HL-1 cells was analyzed using the Dead End Fluorometric TUNEL System (Promega #G3250) according to the manufacturer's instructions. Signals of dead cells were visualized using confocal microscopy 32.

### Cell culture

HL-1 cells were seeded in 6-well plates at a density of 20,000 cells/cm² and cultured in DMEM containing 10% FBS, 1% Hepes, and 1% Penicillin-Streptomycin. To mimic cardiac I/R injury *in vitro*, cells were treated with 0.3 mM hydrogen peroxide for 12 hours. Prior to this treatment, HL-1 cells were incubated with 1-5 mM NR for 6 hours.

### Statistical analysis

Data were analyzed using Prism 8.0 software (GraphPad) and presented as means ± SEM. A two-tailed unpaired Student's t-test was used for comparing two groups, while one-way ANOVA with Tukey's post-hoc tests was used for multiple group comparisons. A p-value <0.05 was considered statistically significant.

## Results

### NR Improves Heart Function After Myocardial Ischemia-Reperfusion (I/R) Injury

**Figure 1:**
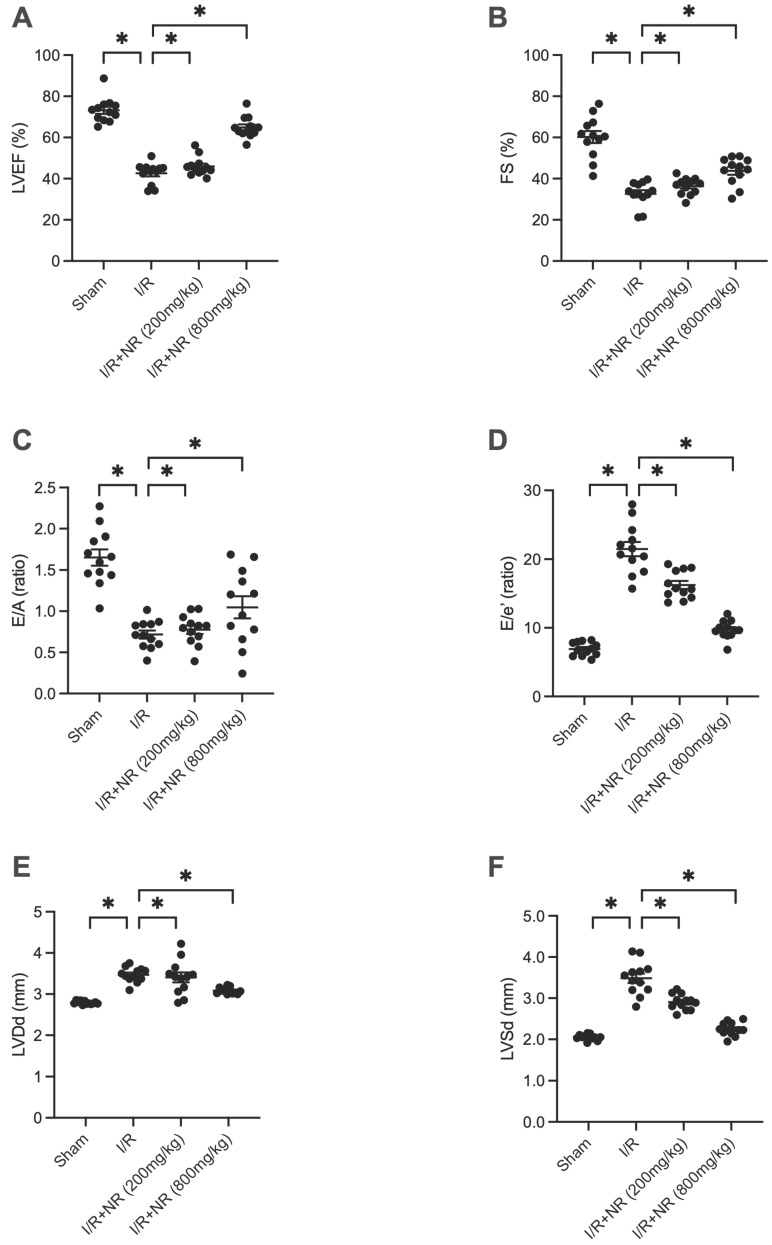
** NR Improves Heart Function After Myocardial Ischemia-Reperfusion (I/R) Injury.** Heart function was evaluated using echocardiography in mice subjected to I/R injury and treated with various doses of Nicotinamide Riboside (NR). **(A)** Left ventricular ejection fraction (LVEF) and (B) fractional shortening (FS) were significantly reduced in the I/R group compared to the sham group, indicating impaired heart function. **(C)** The E/A ratio and **(D)** E/e' ratio, indicators of diastolic function, were also diminished in the I/R group. **(E)** Left ventricular end-diastolic diameter (LVDd) and (F) left ventricular end-systolic diameter (LVSd) were enlarged in response to I/R injury. NR administration significantly improved these parameters in a dose-dependent manner, suggesting its potential therapeutic benefit in mitigating I/R-induced cardiac dysfunction. *p<0.05 indicates a statistically significant difference compared to the sham group.

To assess the role of Nicotinamide Riboside (NR) in protecting the heart against ischemia-reperfusion (I/R) injury, we administered various doses of NR to mice prior to inducing I/R injury. Heart function was subsequently evaluated using echocardiography. As illustrated in Figures 1A-F, I/R injury significantly impaired heart function compared to the sham group, evidenced by reduced left ventricular ejection fraction (LVEF) and fractional shortening (FS). Furthermore, the left ventricular end-diastolic diameter (LVDd) and left ventricular end-systolic diameter (LVSd) were both enlarged in response to I/R injury. The E/A ratio and E/e' ratio were also diminished in mice suffering from I/R injury. Notably, NR administration improved heart function in a dose-dependent manner, suggesting its potential therapeutic benefit in mitigating I/R-induced cardiac dysfunction.

### NR Reduces I/R-Mediated Cardiomyocyte Apoptosis *In Vivo* and *In Vitro*

**Figure 2:**
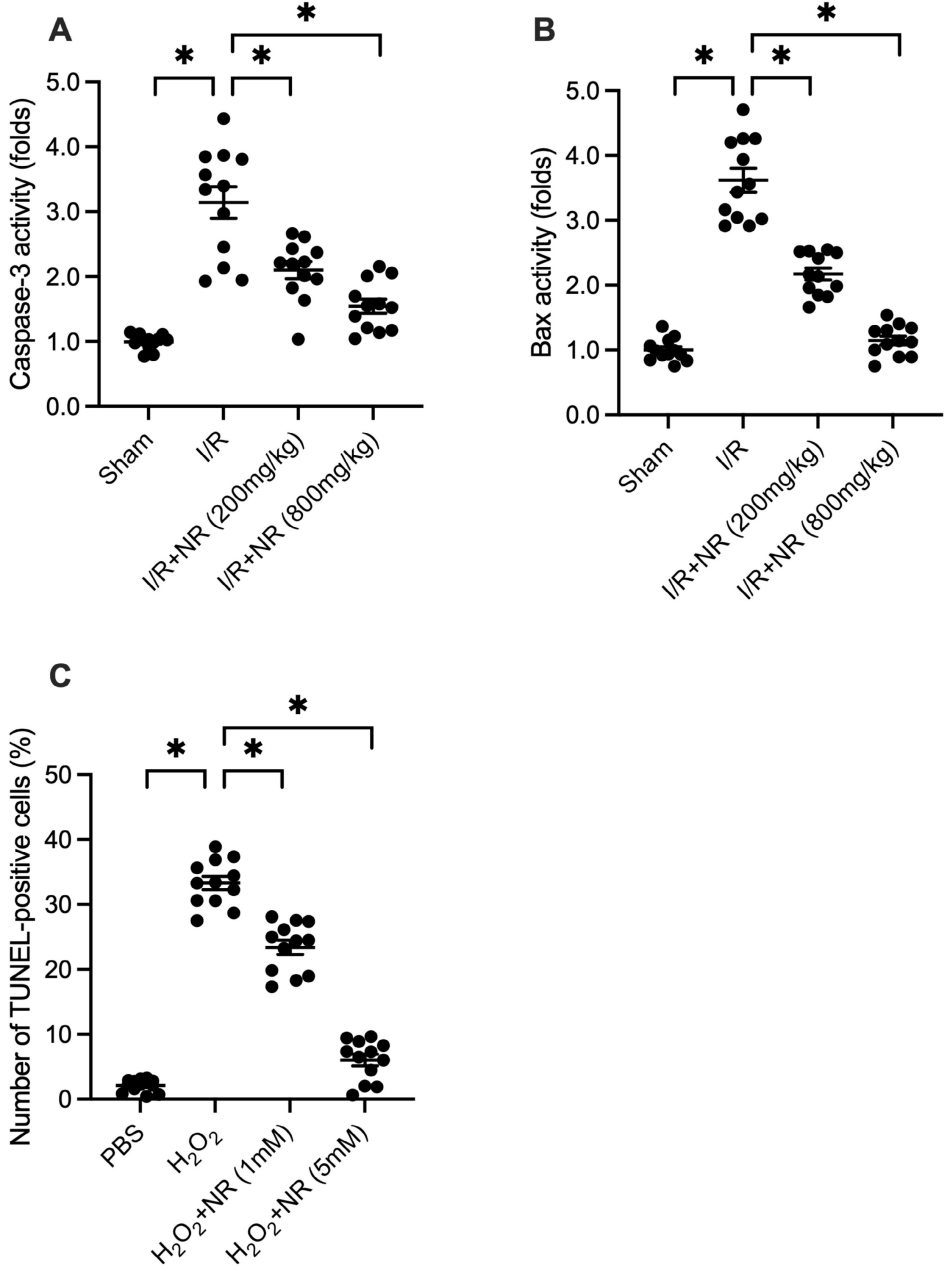
** NR Reduces I/R-Mediated Cardiomyocyte Apoptosis *In Vivo* and *In Vitro*. (A)** Caspase-3 activity, a marker of apoptosis, was significantly elevated in the I/R group compared to the sham group. NR treatment dose-dependently inhibited this increase, as measured by ELISA. **(B)** The pro-apoptotic protein Bax was upregulated in response to I/R injury but returned to physiological levels with NR treatment. **(C)** *In vitro* experiments using HL-1 cells treated with 0.3 mM hydrogen peroxide (H_2_O_2_) to mimic I/R injury showed that NR administration significantly reduced the apoptotic rate from approximately 33% to 8%, as determined by TUNEL staining. These findings collectively indicate that NR markedly reduces cardiomyocyte apoptosis both *in vivo* and *in vitro*. *p<0.05 indicates a statistically significant difference compared to the I/R group without NR treatment.

Cell death is a core mechanism underlying myocardial damage following I/R injury. To investigate whether NR can prevent I/R-mediated cardiomyocyte apoptosis, proteins were isolated from reperfused hearts and analyzed using an ELISA kit to measure caspase-3 activity. As shown in Figure 2A, caspase-3 activity was significantly elevated in the I/R group compared to the sham group, while NR treatment dose-dependently inhibited this increase. Similarly, the pro-apoptotic protein Bax was upregulated in response to I/R injury but returned to physiological levels with NR treatment (Figure 2B). *In vitro* experiments using HL-1 cells treated with 0.3 mM hydrogen peroxide (H_2_O_2_) to mimic I/R injury revealed that NR administration significantly reduced the apoptotic rate from approximately 33% to 8%, as determined by TUNEL staining (Figure 2C). These findings collectively indicate that NR markedly reduces cardiomyocyte apoptosis both *in vivo* and *in vitro*.

### NR Regulates the SIRT3/mtROS/JNK Pathway

**Figure 3:**
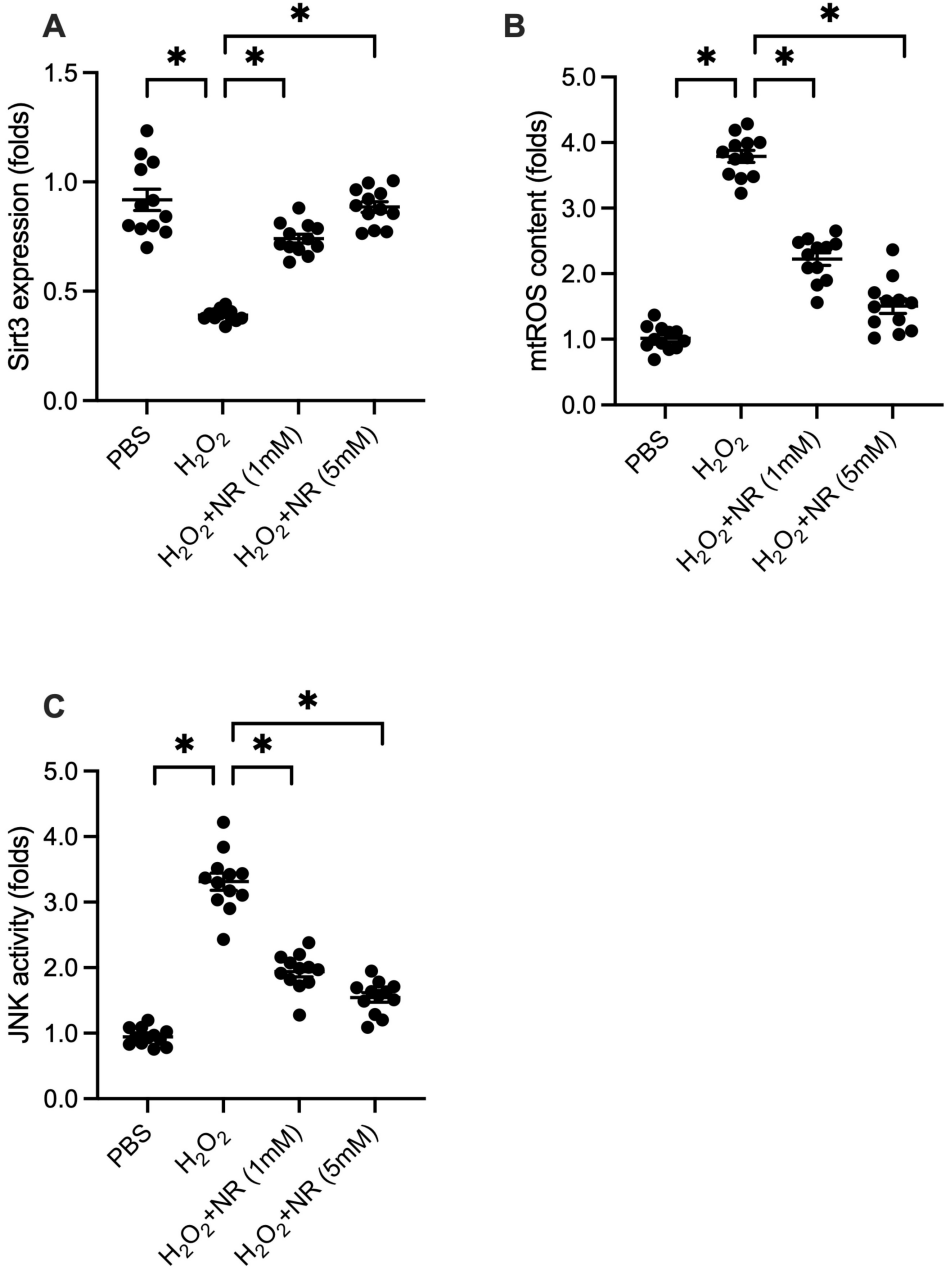
** NR Regulates the SIRT3/mtROS/JNK Pathway. (A)** Western blot analysis showed that SIRT3 expression was significantly downregulated following H_2_O_2_ treatment in HL-1 cells. NR treatment reversed this downregulation, restoring SIRT3 levels. **(B)** Immunofluorescence assays demonstrated a significant increase in mitochondrial reactive oxygen species (mtROS) production in response to H_2_O_2_, which was reduced by NR treatment. **(C)** ELISA results showed that H_2_O_2_ significantly elevated JNK activity, which was abolished by NR treatment. These results demonstrate that NR reverses the downregulation of SIRT3, preventing mtROS accumulation and JNK activation in H_2_O_2_-treated cardiomyocytes. *p<0.05 indicates a statistically significant difference compared to the H_2_O_2_-treated group without NR.

SIRT3 is a potential downstream effector of NR. We explored whether the cardioprotective effects of NR are associated with SIRT3. Western blot analysis of proteins isolated from HL-1 cells showed that SIRT3 expression was significantly downregulated following H_2_O_2_ treatment (Figure 3A). However, NR treatment reversed this downregulation, restoring SIRT3 levels. SIRT3 is known to play a role in maintaining mitochondrial redox balance by promoting antioxidant signaling. We examined whether increased SIRT3 expression was associated with decreased mitochondrial reactive oxygen species (mtROS) production. Immunofluorescence assays demonstrated a significant increase in mtROS production in response to H_2_O_2_, which was reduced by NR treatment (Figure 3B). Furthermore, previous studies have identified mtROS as an upstream activator of the JNK pathway, a pro-apoptotic factor in cardiomyocytes. ELISA results showed that H_2_O_2_ significantly elevated JNK activity (Figure 3C), which was abolished by NR treatment. Our results demonstrate that NR reverses the downregulation of SIRT3, thereby preventing mtROS accumulation and JNK activation in H_2_O_2_-treated cardiomyocytes.

### Cardioprotective Effects of NR Are Abrogated in SIRT3-Knockout Mice

**Figure 4:**
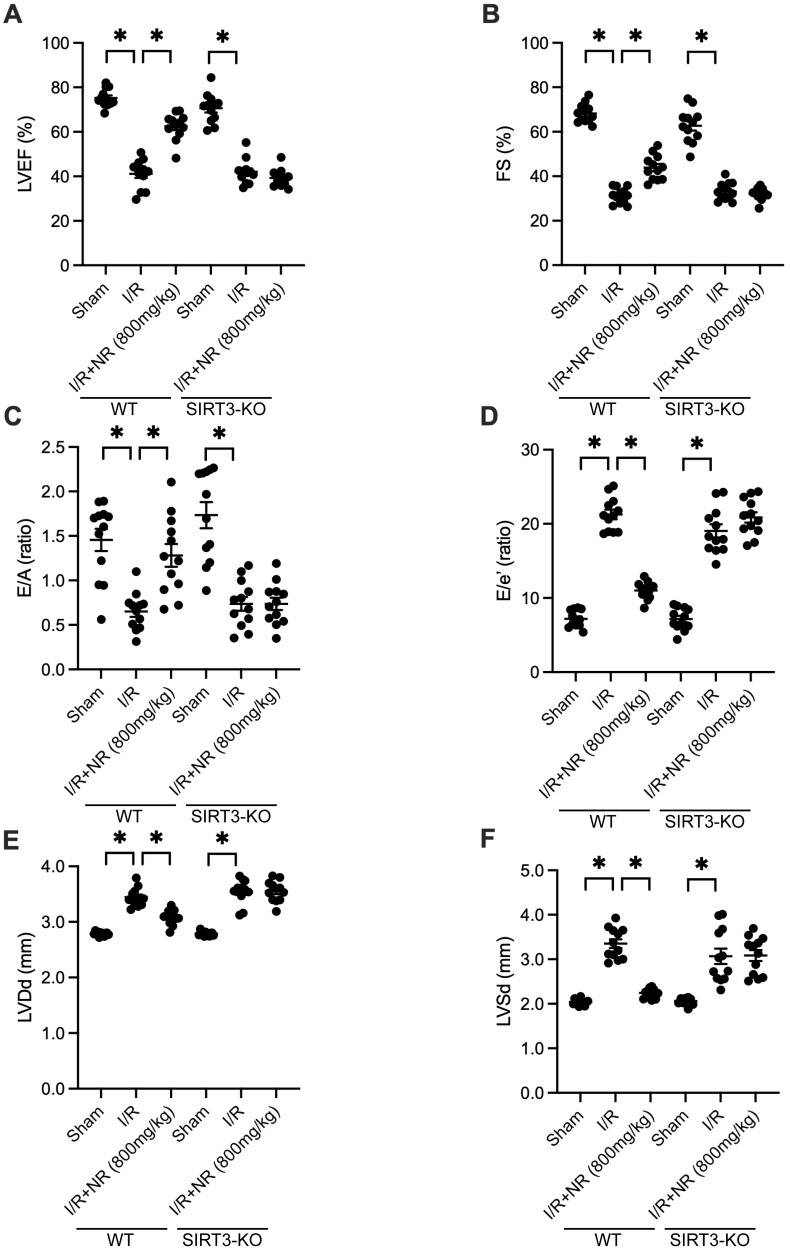
** Cardioprotective Effects of NR Are Abrogated in SIRT3-Knockout Mice.** Heart function was evaluated using echocardiography in both wild-type (WT) and SIRT3-knockout (SIRT3-KO) mice subjected to I/R injury. **(A)** Left ventricular ejection fraction (LVEF) and **(B)** fractional shortening (FS) were impaired in WT mice following I/R injury, but NR treatment improved these parameters. In SIRT3-KO mice, the cardioprotective effects of NR were abrogated, as evidenced by the lack of improvement in LVEF and FS. **(C)** The E/A ratio and **(D)** E/e' ratio, which were normalized by NR in WT mice, were not improved in SIRT3-KO mice. **(E)** Left ventricular end-diastolic diameter (LVDd) and **(F)** left ventricular end-systolic diameter (LVSd) were not improved in SIRT3-KO mice. These findings suggest that the cardioprotective effects of NR are highly dependent on the presence of SIRT3. *p<0.05 indicates a statistically significant difference compared to the WT I/R group without NR treatment.

To determine whether SIRT3 is essential for NR-mediated cardioprotection, we utilized SIRT3-knockout (SIRT3-KO) mice. Echocardiography was performed to measure heart function. As shown in Figures 4A-F, I/R injury impaired heart function in wild-type (WT) mice, but this impairment was mitigated by NR. However, in SIRT3-KO mice, the cardioprotective effects of NR were abrogated, as evidenced by blunted LVEF and FS. Additionally, NR failed to improve LVDd and LVSd in SIRT3-KO mice. Relaxation parameters, such as the E/A and E/e' ratios, which were normalized by NR in WT mice, were not improved in SIRT3-KO mice. These findings suggest that the cardioprotective effects of NR are highly dependent on the presence of SIRT3.

## Discussion

The present study has identified several significant findings regarding the protective effects of Nicotinamide Riboside (NR) against myocardial ischemia-reperfusion (I/R) injury. Our results demonstrate that NR significantly improves heart function post-I/R injury, reduces cardiomyocyte apoptosis, and modulates key signaling pathways involved in oxidative stress and cell death. Specifically, NR's ability to enhance SIRT3 expression, reduce mitochondrial reactive oxygen species (mtROS), and inhibit the c-Jun N-terminal kinase (JNK) pathway highlights its innovative potential. These findings suggest that NR could be developed as a novel therapeutic agent for the treatment of ischemic heart diseases, offering a new approach to mitigating I/R-induced cardiac damage and improving clinical outcomes.

Nicotinamide Riboside has shown protective effects in various cardiovascular diseases beyond myocardial I/R injury 33, 34. NR supplementation boosts NAD^+^ levels, which are critical for cellular metabolism and repair processes 35, 36. Recent animal studies have demonstrated that NR can reduce infarct size, improve cardiac function, and decrease oxidative stress markers 37-40. Additionally, preliminary clinical trials have indicated that NR is well-tolerated and effectively increases NAD^+^ levels in humans 41. These findings underscore the potential of NR as a therapeutic agent not only for I/R injury but also for other cardiovascular conditions, such as heart failure and cardiomyopathies, highlighting its broad clinical applicability 42-44.

In recent years, research on myocardial ischemia-reperfusion (I/R) injury has made significant strides, particularly in understanding the molecular and cellular mechanisms underlying this condition 45, 46. Key areas of focus have included the role of oxidative stress, inflammation, and apoptosis in exacerbating myocardial damage post-reperfusion 47-49. Studies have highlighted the importance of mitochondrial function and redox balance in mitigating I/R injury 50-53. The relevance of our study lies in its exploration of Nicotinamide Riboside (NR) as a modulator of these critical pathways. By enhancing NAD^+^ levels and activating SIRT3, NR addresses the oxidative stress and mitochondrial dysfunction central to I/R injury. Furthermore, our findings contribute to the growing body of evidence supporting the therapeutic potential of targeting mitochondrial pathways and oxidative stress in I/R injury, reinforcing the need for innovative strategies like NR supplementation in clinical settings.

SIRT3, a mitochondrial deacetylase, plays a crucial role in protecting the heart against I/R injury by maintaining mitochondrial integrity and function 54, 55. Our study confirms that NR-mediated cardioprotection is associated with the upregulation of SIRT3, which enhances the activity of antioxidant enzymes, thereby reducing mtROS production. Previous studies have shown that increased SIRT3 activity is essential for reducing oxidative stress and preventing mitochondrial dysfunction during I/R injury 56-58. Additionally, mtROS is a known upstream activator of the JNK pathway, which induces apoptosis in cardiomyocytes 59. By reducing mtROS levels, NR effectively inhibits JNK activation, further protecting the heart from I/R-induced cell death. This study integrates and extends previous research on the SIRT3/mtROS/JNK signaling axis, providing a comprehensive understanding of its role in myocardial protection.

While most of the research on NR has been conducted in preclinical models, there is emerging evidence supporting its potential application in clinical settings. Initial clinical trials have demonstrated that NR supplementation is safe and well-tolerated in humans 41, effectively raising NAD^+^ levels without significant adverse effects. These trials have shown promise in improving metabolic health and reducing biomarkers of oxidative stress and inflammation, which are relevant to cardiovascular diseases. However, the direct application of NR in patients with myocardial I/R injury is still in its early stages. Further large-scale, randomized controlled trials are necessary to establish the efficacy and safety of NR in this specific patient population. The positive outcomes observed in preclinical studies provide a strong rationale for advancing clinical research to fully explore NR's therapeutic potential in protecting against myocardial I/R injury and other cardiovascular conditions.

While our findings provide significant insights into the protective mechanisms of NR, several limitations should be acknowledged. Firstly, our study primarily utilized animal and cell models, which may not fully replicate the complexity of human myocardial I/R injury. Therefore, the translation of these findings to clinical practice requires further validation through extensive human clinical trials. Secondly, although we demonstrated the involvement of the SIRT3/mtROS/JNK pathway in NR-mediated cardioprotection, a more in-depth investigation into the molecular mechanisms is needed to fully elucidate the precise regulatory interactions. Addressing these limitations in future research will be critical for advancing NR as a viable therapeutic option for myocardial I/R injury and other cardiovascular diseases.

In conclusion, this study highlights the cardioprotective effects of Nicotinamide Riboside through the modulation of the SIRT3/mtROS/JNK signaling pathways. By demonstrating NR's potential to alleviate myocardial I/R injury, our research paves the way for future clinical applications and underscores the need for further studies to validate and expand upon these promising findings.
